# Role modelling in the training of hospital-based medical specialists: a validation study of the Role Model Apperception Tool (RoMAT)

**DOI:** 10.1007/s40037-019-00527-6

**Published:** 2019-07-25

**Authors:** Miran Said, Ria H. G. A. Jochemsen-van der Leeuw, Bea Spek, Paul L. P. Brand, Nynke van Dijk

**Affiliations:** 1 Department of General Practice/Family Medicine, Amsterdam UMC, location AMC, University of Amsterdam, Amsterdam, The Netherlands; 2 Department of Clinical Epidemiology, Biostatistics and Bioinformatics, Amsterdam UMC, location AMC, University of Amsterdam, Amsterdam, The Netherlands; 3 Isala Hospital, Zwolle, The Netherlands; 4 UMCG Postgraduate School of Medicine, University of Groningen and University Medical Center Groningen, Groningen, The Netherlands

**Keywords:** Education, Internship and Residency, Role modelling, Clinical teacher

## Abstract

**Introduction:**

Role modelling is a key component in the training of doctors that influences professional behaviour, identity and career choices. Clinical teachers and residents are often unaware of this, thereby risking transmission of negative behaviour. On the other hand, awareness positively affects role model behaviour. To assess role model behaviour, the Role Model Apperception Tool (RoMAT) was developed and validated in general practice training. The aim of the current study was to validate the RoMAT in the hospital-based training setting.

**Methods:**

The authors asked first to last year residents, regardless of their specialty, to participate after written approval from their clinical teachers. The tool was completed online in 2017. The authors performed a principal component analysis and investigated internal consistency, construct validity, inter-rater reliability, known-groups comparisons and floor and ceiling effects.

**Results:**

Of the 473 residents contacted, 187 (40%) completed the questionnaire. As in the primary validation study, the authors extracted two components: ‘Caring Attitude’ and ‘Effectiveness’, explaining 67% of the variation with a Cronbach’s alpha of 0.94 and 0.93 respectively. Evidence for construct validity was found and there were no floor or ceiling effects, but inter-rater reliability was low.

**Discussion:**

The RoMAT was internally consistent and valid to assess role model behaviour of the clinical teacher towards the resident in the hospital-based training of medical specialists. The poor inter-rater reliability, most likely due to homogeneous RoMAT responses, should be borne in mind when evaluating RoMAT scores on individual clinical teachers.

**Electronic supplementary material:**

The online version of this article (10.1007/s40037-019-00527-6) contains supplementary material, which is available to authorized users.

## What this paper adds

Role modelling is a very powerful concept in medical education and is present in all phases of training. It is a dynamic process that influences young doctors and shapes careers. Awareness of this process is critical. This paper builds upon previous research and is the first to validate a tool to assess role modelling in the clinical training setting. We found support for the validity and internal consistency of the tool; however, inter-rater reliability scores were low. This should be borne in mind when using this tool in clinical practice.

## Introduction

Medical education largely takes place in clinical practice where clinical teachers play a pivotal role [1, 2]. Evidence supporting this emerged in the previous century, when the social learning theory was developed. This theory states that learning is a cognitive process in a social setting where behaviour can be learned through observation. Imitativeness, a component of behavioural learning, consists of an individual imitating a behaviour consciously depending on the response the behaviour evoked [3]. Albert Bandura demonstrated through a series of experiments the importance and effectiveness of modelling for attaining new behaviour [4, 5]. In medical education, workplace-based learning forms an important component of the training program and therefore should be used effectively [6]. Role modelling is a key component of this, which fits the framework of the social learning theory[7] and has been described in the literature as the process in which ‘faculty members demonstrate clinical skills, model and articulate expert thought processes and manifest positive professional characteristics’ [1]. The resident is influenced, positively or negatively, by the clinical teacher through his or her behaviour during daily practice [1, 7–10]. An individual is a positive role model when he or she exhibits excellent teaching skills, clinical skills and personal factors such as compassion, integrity and honesty [2]. Role modelling is a dynamic process where the resident observes, judges and consciously and subconsciously decides if what is observed will be implemented into a personal style [10]. Role modelling exists in all phases of medical training and influences professional behaviour and identity and shapes career choices, indicating its power [11–13]. Interestingly, clinical teachers and residents are not always aware of this process, risking transmission of negative behaviour [14–16]. Awareness of being a role model improves role model behaviour [17]. For this reason, Jochemsen-van der Leeuw et al. identified characteristics of role model behaviour [11], and developed and subsequently validated the Role Model Apperception Tool (RoMAT) in the primary care training setting [18]. The purpose of this tool is to help residents assess positive and negative role modelling and serve as feedback to the clinical teacher on role model behaviour.

In the Netherlands, postgraduate medical education in primary care and secondary (hospital-based) care is arranged differently. Training takes 3 years in primary care and 5–6 years in secondary care. In primary care training, supervision in patient care work is usually done by a single general practitioner teacher and healthcare is provided locally in the community (2100 patients per full-time general practitioner). In the training of medical specialists in secondary care, supervision is more fragmented, with clinical teachers supervising multiple residents simultaneously, and residents being supervised by multiple clinical teachers. Moreover, healthcare is given on a larger scale in hospitals. These differences in the context of training may cause variation in the way role model behaviour is expressed by clinical teachers and perceived by residents in primary versus secondary care.

Currently no tool is used to assess the various components of role modelling as a specific entity in the training of medical specialists. Instruments to assess the quality of hospital-based clinical teachers are being used and contain some items on role modelling, but lack specificity to identify important characteristics of good or bad role models [19]. Furthermore we do not know whether observations on these characteristics differ per trainee. Workplace-based learning is interactive and based on observation and imitation[6] (as with role modelling), and behaviour, good or bad, is transmitted consciously and subconsciously [14–16]. We believe that to improve role modelling[17] and to avoid transmission of negative behaviour, awareness of role modelling in the training of medical specialists must be enhanced. The intended use of the RoMAT for this new context is to help residents assess role model behaviour (good and bad), to serve as feedback to clinical teachers and to compare clinical teachers regarding role model behaviour.

Psychometric tool characteristics, such as validity, are not merely a reflection of the tool alone, but are also dependent of the context of application [20–23]. We therefore aimed to validate the RoMAT and to investigate the possibility to compare clinical teachers regarding role model behaviour in the hospital-based training setting.

## Methods

### Population and design

We performed this study in hospital-based training settings and included residents supervised by a clinical teacher, regardless of specialty or year of training. We contacted clinical teachers from Dutch hospitals, academic and general teaching hospitals, by email for approval to approach their residents. After receiving approval, we sent residents instructions and asked for voluntary participation. Online they could fill in one RoMAT per clinical teacher.

Participants gave informed consent and were coded to make sure responses were untraceable. The encryption of the names and codes was stored safely and was not accessible for other researchers after sending the invitation. Clinical teachers could request their average RoMAT score, as given by residents, making sure that study and ‘real world’ context were alike, ensuring that psychometric characteristics reflect future use [20, 21]. We raffled a small incentive (a 50 euro Restaurant Gift Voucher) in a lottery. We received ethical approval from the Dutch Association of Medical Education (NVMO, NERB file no. 852).

### The RoMAT

The RoMAT consists of 17 five-point Likert scale items (one = total agreement, five = total disagreement). These items reflect three qualities (clinical, teaching and personal) and were derived from characteristics of role models [11]. Face and content validity were ensured with experts reviewing the items and with a pilot test. A principal component analysis (PCA) revealed two components: ‘Caring Attitude’ and ‘Effectiveness’. The former includes items that reflect ‘characteristics of the relationship of clinical teachers to their patients, residents, and others’, and the latter represents items relating to ‘the ability of clinical teachers to provide their patients and residents with what they need.’ These factors explained 57% of the variance of responses within the dataset and both were internally consistent. Moreover, the tool proved to be construct valid [18].

For the current study, residents filled in the RoMAT and gave information regarding themselves, their clinical teacher and the context of training such as age, gender, specialty and year of training. They could give feedback to the primary researcher about the RoMAT through email. We excluded responses with missing values in the RoMAT (listwise deletion). We selectively excluded participants with missing data in questions regarding personal, clinical teacher and context characteristics in analysis regarding this information, while keeping their RoMAT for analysis.

### Validation

We assessed internal consistency by calculating Cronbach’s alpha of the dimensions derived by PCA and quantified interrelatedness of items [24]. Values <0.7 suggest heterogeneity in items, whereas values >0.9 indicate homogeneity or redundant items [25]. Bartlett’s test helped to identify sufficient variable correlation for PCA and if significant indicated that the correlation matrix is not an identity matrix. We investigated multicollinearity by inspecting the correlation plot and matrix, item-total correlations and the determinant of the R‑matrix [26, p. 75, 26, p. 81]. We calculated the Kaiser-Meyer-Olkin (KMO) measure and the participant-to-item ratio for sampling adequacy. A KMO value >0.8 and a ratio >10:1 with an absolute number of participants greater than 100 were deemed to be excellent [27, 28].

For this study we sought the find evidence of construct validity which is assessed when a gold standard is lacking and refers to the degree to which the scores of a measurement instrument are consistent with hypothesis. The extent to which scores on an instrument reflect the dimensionality of the construct adequately is defined as structural validity [24]. First of all we investigated this with a PCA. This gave insight into interactions between items, the underlying tool structure and concordance with the previous study regarding both components [18]. We extracted components based on the underlying theory and the Kaiser criterion (inclusion of factors with an eigenvalue >1) and were aided by making a scree plot with a parallel analysis, a robust method to empirically determine the optimal number of factors in a PCA [29]. As a correlation between both factors was expected, we used oblique rotation for enhanced interpretation of the components. Secondly, we assessed construct validity by calculating the correlation between the RoMAT and a five-point Likert scale question: ‘Do you consider your clinical teacher to be a good role model?’ and hypothesized a moderate to high (Spearman *r* > 0.40) correlation [30]. Finally, we assessed differences in scores between male and female [18, 31, 32] and more and less experienced residents [18, 33]. In line with previous studies we hypothesized that there would be no differences in scores between male and female residents[18, 32] and that less experienced residents would score lower (better role model behaviour) on items related to the ‘Caring Attitude’ component [18].

### Reliability

In order to investigate if the RoMAT could be used to compare clinical teachers as role models, we calculated the intraclass correlation coefficient (ICC) as a measure of inter-rater reliability (IRR) and used a one-way random approach [34]. Due to the unequal number of observations per clinical teacher, we randomly selected three observations for each. We excluded clinical teachers with less than three observations for ICC calculations. We considered values greater than 0.75 to be excellent [35].

### Floor and ceiling effects

Floor and ceiling effects were present if more than 15% of the participants scored the highest or lowest possible score on the questionnaire. When present, reliability may be affected due to the inability to distinguish responses within these lowest and highest scoring categories [36].

### Statistical analysis

We used statistical software R for Windows version 3.2.5 (R Core Team, Vienna, Austria; 2017) and RStudio for Windows version 1.0.143 (RStudio, Inc, Boston, USA, 2016) for analysis. Demographic characteristics are displayed as frequency with percentage, and as mean with standard deviation (SD) or median with interquartile range. RoMAT scores per group are presented as mean and SD [37]. We set a two-tailed significance level of 0.05 for all tests.

## Results

### Response

**Fig. 1:**
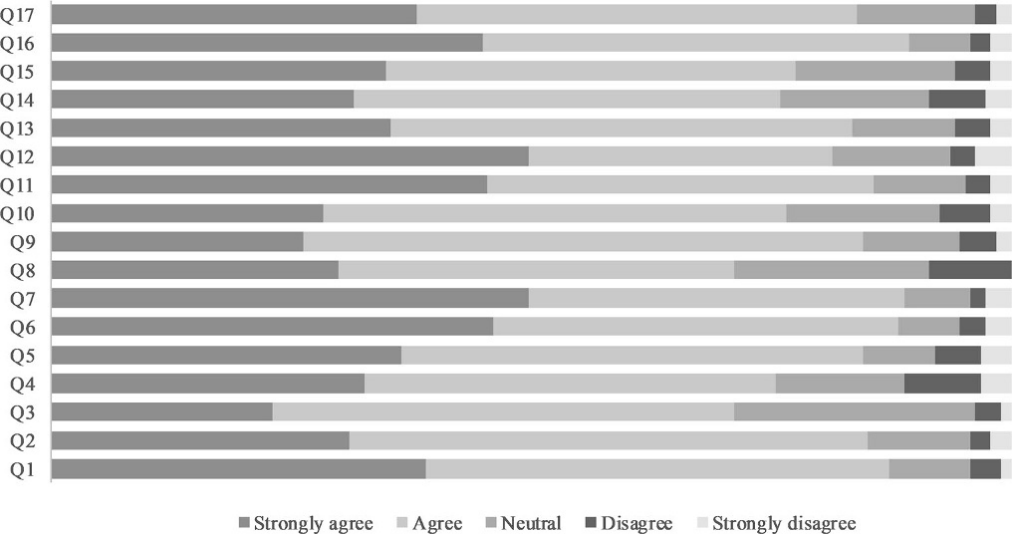
Bar chart. Distribution of chosen score categories by residents on the 5‑point Likert-scale of the RoMAT for each question

Of the 473 residents contacted, 188 completed the questionnaire. One participant’s questionnaire was discarded as it was filled in wrongly due to a technical issue. The remaining 187 residents (response rate 40%) were from 20 specialties in five hospitals, assessing 35 clinical teachers. Seven cases showed major discrepancies in RoMAT entries and the single role model question, likely caused by misinterpretation of the Likert scale. To prevent over-interpretation, however, we kept these responses unchanged, although post-hoc analyses to assess its influence were performed (see below). Feedback given through email was that some items regarding interaction with patients were not fully applicable to some medical specialties such as medical microbiology and radiology. Demographic characteristics are presented in the Table in the online Supplementary Electronic Material. Responses for each item are shown in Fig. 1.

### Validation

The correlation matrix and plot demonstrated sufficient correlations, with none being less than 0.20 or greater than 0.90. The determinant of the R‑matrix was 7.49E^−07^ indicating a risk of multicollinearity. With a maximum inter-item correlation of 0.79 (94% of all correlations <0.70) [26, p. 81] and item-total correlations between 0.54–0.81, this was accepted. Sample size was adequate, with a participant-to-item ratio of 11:1, an absolute number of participants >100 and a KMO of 0.94 for the total scale and 0.90–0.97 per item. A significant Bartlett’s test (*p* < 0.001) allowed us to perform PCA. Internal consistency was high (Cronbach’s alpha = 0.93/0.94 for both factors (mentioned later)).

**Fig. 2:**
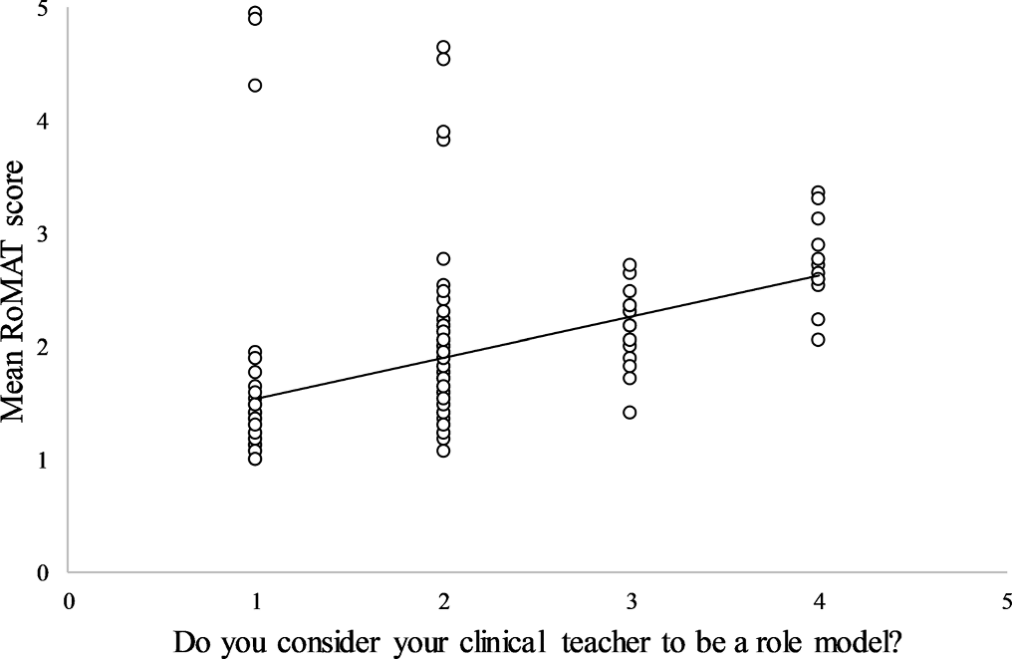
Strip chart, showing the relation between the single role model question and the mean RoMAT score (construct validity). Lower scores indicate better role model behaviour. Evident are the seven outliers

The parallel analysis demonstrated that a one factor structure was optimal. However, based on the Kaiser criterion and supported by the underlying theory from the primary study[18], stating that the tool consists of two factors, ‘Caring Attitude’ and ‘Effectiveness’, we extracted two components (Tab. 1). After oblique rotation, factor 1 and 2 had an eigenvalue of 6.67 and 4.69 respectively and together explained 67% of the variance. Fifteen items belonged to the same factors as in the previous study. All items, except item 7, had a unique loading of ≥0.40. This combined with a fit based upon of diagonal values of 0.99 was indicative for a good fit of the model and construct validity. Correlation between the single role model question and the RoMAT questionnaire was strong (Spearman* r* = 0.62) (Fig. 2). Residents with more previous experience tended to give lower scores (better role model behaviour) than residents with less previous experience on the ‘Effectiveness’ component (*p* = 0.03, Spearman* r* = −0.15). A statistically significant difference in scoring on the ‘Caring Attitude’ component regarding year of training was found (*p* = 0.05). No gender influences on scores were found (Tab. 2).

**Table 1:** Exploratory factor analysis of the RoMAT with oblique rotated factor loadings^a,b^ (*n* = 187)

	Factor 1c	Factor 2c	Communalities
**My clinical teacher:**			
Conveys empathy for patients (item 2)	**0.73**	0.12	0.65
Communicates well with patients and relatives (item 3)	**0.73**	0.04	0.58
Establishes rapport with learners (item 5)	**0.82**	0.06	0.73
Has a positive attitude towards learners (item 6)	**0.72**	0.20	0.73
Is patient (item 8)	**0.94**	−0.33	0.61
Has a positive interaction with other health care workers (item 9)	**0.67**	0.20	0.65
Is available for learners (item 12)	**0.62**	0.22	0.60
Is honest and has integrity (item 13)	**0.71**	0.16	0.67
Is nice and easy to work with (item 16)	**0.86**	0.04	0.78
Is professionally competent in difficult clinical situations and able to cope with adversity (item 17)	**0.53**	0.38	0.66
Has excellent clinical reasoning skills (item 1)	0.12	**0.70**	0.61
Understands learners’ needs and is committed to the growth of learners (item 4)	0.36	**0.51**	0.61
Makes learning exciting and stimulating (item 10)	0.16	**0.74**	0.73
Has self-confidence (item 11)	0.08	**0.80**	0.72
Has leadership qualities (item 14)	−0.12	**0.94**	0.76
Is aware of his/her role model status (item 15)	0.17	**0.68**	0.63
Demonstrates enthusiasm for his/her work (item 7)	*0.44*	*0.46*	*0.65*
Eigenvalue	6.67	4.69	–
Variance explained	39%	28%	–
Cronbach’s alpha	0.94	0.93	–

^a^Factor loadings ≥0.5 are in bold print

^b^Cross loadings are in italic print

^c^Factor 1 and Factor 2 are titled ‘Caring Attitude’ and ‘Effectiveness’ respectively

**Table 2:** Known-groups comparisons of residents

	‘Caring Attitude’		‘Effectiveness’	
**Groups**	Mean (SD)	*p*-value	Mean (SD)	*p*-value
**Gender**		0.49^*^		0.77^*^
*Male*	1.87 (0.74)		1.98 (0.85)	
*Female*	1.90 (0.70)		1.87 (0.69)	
**Year of training** ^a^		0.05^b^		0.25^b^
*1*	1.86 (0.78)		1.83 (0.90)	
*2*	1.91 (0.64)		1.84 (0.62)	
*3*	2.16 (1.00)		2.03 (1.03)	
*4*	1.80 (0.53)		1.88 (0.55)	
*5*	1.98 (0.45)		2.10 (0.58)	
*6*	1.44 (0.32)		1.91 (0.69)	
*Not in training*	1.53 (0.45)		1.65 (0.56)	
**Experience** ^c,d^		0.83^e^		0.03^e^

*Mann-Whitney U test used for statistical analysis

^a^Two residents were excluded due to missing data regarding this question

^b^Kruskal-Wallis test used for statistical analysis

^c^Experience as a resident prior to current function in months

^d^Analyzed as continuous variable

^e^Spearman Rank correlation test used for statistical analysis

### Reliability

Inter-rater reliability calculated for 23 clinical teachers with a minimum of three scores was inadequate (ICC = 0.08, 95% CI = −0.14–0.37). Removal of data regarding the seven residents who presumably misunderstood the Likert scale yielded a similar result (ICC = 0.07, 95% CI = −0.06–0.47).

### Floor and ceiling effects

With 2% of the participants giving the lowest (good role model), and none giving the highest (bad role model) possible score, we detected no floor or ceiling effects.

## Discussion

We validated the RoMAT in the training of medical specialists. The items of the tool were internally consistent and supporting evidence for construct validity was found. However, the ICC was low. Through PCA we extracted two components explaining 67% of the variation: ‘Caring Attitude’ and ‘Effectiveness’.

Compared with the primary study, one difference was the method of administration (paper-based versus web-based). Previous studies showed no differences in psychometric properties between paper-based and web-based administration of health measuring tools [38, 39]. We therefore believe that the method of administration did not influence our results. An advantage is that web-based surveys can reduce the risk of missing or ambiguous data by allowing one option to be chosen and prohibiting participants from proceeding when an item is not filled in (SurveyMonkey Inc., San Mateo, CA, USA).

In spite of the scree plot with a parallel analysis suggesting a one component structure, we extracted two components based on the underlying theory from the primary study. With eigenvalues greater than one, this was in accordance with the Kaiser criterion [40]. ‘Caring Attitude’ is defined as ‘the cluster of items reflecting relationship characteristics between clinical teachers to their patients, residents and others’, whereas ‘Effectiveness’ is defined as ‘the cluster of items relating to the ability of clinical teachers to provide their patients and residents with what they need’. Compared with the primary study, both components consisted of the same items, except for two [18]. Firstly item 7: ‘my clinical teacher demonstrates enthusiasm for his work’ fitted both components. We did not omit this item as it addresses a key aspect of role modelling [41]. Secondly, item 17: ‘my clinical teacher is professionally competent in difficult clinical situations and able to cope with adversity’, fitted the ‘Caring Attitude’ component in our study whereas it belonged to the ‘Effectiveness’ component in the primary study [18]. We found that this item did not seem to fit both components very well, as is illustrated by its low rotated factor loadings (Tab. 1). In the previous study low rotated factor loadings were also found [18]. This bad fit of item 7 and 17 can be explained by the differences between these items and both components. Both items reflect traits of the clinical teacher that are very personal in nature. On the other hand, both components are defined to reflect characteristics of the interaction between the clinical teacher and his/her personal environment. This discrepancy could explain our results. The good accordance between both studies regarding the underlying structure of the tool supports its construct validity and could imply that role model behaviour expressed by clinical teachers and perception of this by residents might be similar in primary care and secondary care training settings.

Internal consistency was high, reflected by a Cronbach’s alpha 0.94 and 0.93 for factor 1 and 2 respectively. In the literature it has been stated that values >0.9 may signal overlapping and thus redundant items [25], which may have been the case with the RoMAT. However, we have deliberately decided not to omit any items because of the need to address all specific aspects of role modelling reflected by all 17 items of the RoMAT.

We found a strong correlation between the single role model question and the RoMAT (Spearman *r* = 0.62), supporting evidence of construct validity. We found that residents with more prior experience gave lower scores (i.e. better role model behaviour) in the ‘Effectiveness’ component compared with residents with less experience. This is in accordance to previous literature[31, 33]. This correlation was not found in the primary study of the RoMAT[18]. Jochemsen-van der Leeuw studied residents in family medicine, where Côté et al.[42] found that these residents tend to choose role models with a more patient and colleague centred approach to care and focus less on clinical expertise compared with other specialties. This suggests that in hospital-based training characteristics of role models reflecting the ‘Effectiveness’ component are considered more important than characteristics from the ‘Caring Attitude’ component, and may explain this discrepancy. In contrast to the primary study[18] we found that younger year residents (years 1 to 3) gave higher scores (i.e. worse role model behaviour) on the items on the ‘Caring Attitude’ component compared with older year residents (years 4 to 6), although differences were small. This discrepancy might be caused by two factors. Firstly, although hospital-based clinical teachers nowadays become a clinical teacher voluntarily and receive more and better education regarding training, clinical teachers in general practice still undergo more comprehensive and frequent training [43]. Secondly, in hospital a multitude of residents and clinical teachers work at the same time, which makes interaction more fragmentary and less personal compared with training in general practice where supervision is done in a one-on-one fashion most of the time. In our study, this could have caused younger year residents to miss personal attention resulting in higher scores on the ‘Caring Attitude’ component. Moreover, as residents gain more experience this need for attention may be reduced and their focus shifts from personal to professional qualities, causing the ‘Caring Attitude’ scores to be lower for older year residents as they are less affected by fragmentary and less personal supervision. In accordance with previous studies we found no differences between males and females [18, 32].

In the current study, the majority of the scores were in the top three score categories (Fig. 1). Several factors may have caused this. Research has shown that in studies investigating questionnaires or self-reported measures, respondents may give socially desirable answers. Usually this phenomenon is present when participants are asked about sensitive subjects [44]. Moreover, in ‘Agree/Disagree’ formulated questions, respondents may have the tendency to answer Agree regardless of their opinion. This phenomenon is called acquiescence bias and is of uncertain origin [45]. On the other hand, the high and relatively homogeneous scores may well reflect the overall satisfaction of residents with the role model behaviour of their clinical teacher, possibly due to the strong focus on ongoing faculty development and clinical teaching skills of clinical teachers in the Netherlands, which is monitored during 5‑yearly recertification procedures of each training program [46]. In clinimetrics, it has been shown that sufficient variation in responses is necessary to attain high ICC values [47, 48]. We therefore believe that the poor IRR with low ICC are possibly a result of this homogeneity in responses and reflect our study population instead of tool characteristics. Nevertheless, this implies that the RoMAT might not be suitable to compare clinical teachers based on their scores.

Resident training is largely workplace-based[6] and learning occurs through observation and imitation[3] and subsequently through role modelling [10]. Literature has shown that students might benefit from early awareness of role model behaviour and clinical teachers have stated that they rarely receive feedback on the impact of their role modelling and lack awareness [7]. Awareness of role modelling improves role model behaviour [17]. This is where the RoMAT is of great use and fills in the current educational gap: it helps residents gain insight into their needs from their clinical teacher as a role model and serves as a source of feedback to clinical teachers.

### Limitations

Because clinical teachers’ approval to approach their residents was sought and because participation of residents was voluntary, selection of clinical teachers who have better role model behaviour and are open to feedback may have occurred. This could have led to higher scores with less diversity causing lower ICC values [47, 48]. Also, an unequal number of responses from different specialties and individual clinical teachers was present, either increasing or decreasing the overall scores depending on which clinical teachers were overrepresented. Finally, residents from specific medical specialties such as Radiology and Medical Microbiology were sometimes unable to answer specific items regarding interaction with patients (mainly items 2 and 3).

### Future studies

Future research should focus on making minor modifications for specialties with very little interaction with patients, possibly by removal of items 2 and 3 and adding extra items regarding their specialty. Also, before implementing the RoMAT in personal teaching evaluations, intra-rater reliability should be researched. Finally, our results should be confirmed by more powerful analysis such as Item Response Theory methods [26, p. 84–91].

The RoMAT has high internal consistency and sufficient construct validity to be used as a tool to evaluate role model behaviour of hospital-based clinical teachers in the setting of postgraduate medical education. The poor inter-rater reliability found in this study is most likely due homogeneity in responses among residents. This limitation should be borne in mind when evaluating RoMAT scores on individual clinical teachers.

## Caption Electronic Supplementary Material

Supplementary 1: RoMAT

Table 1: Characteristics of residents, clinical teachers and context
