# Supplementary material for: Role modelling in the training of hospital-based medical specialists: a validation study of the Role Model Apperception Tool (RoMAT)
Source: Perspect Med Educ. 2019 Jul 25;8(4):237–45. doi: 10.1007/s40037-019-00527-6 (PMC6684559; doi:10.1007/s40037-019-00527-6)
Supplement: Supplementary file 1 — Supplementary 1: RoMAT [file 40037_2019_527_MOESM1_ESM.docx]

**Supplementary**

**Supplementary 1: RoMAT**

| **Nr** | **My clinical teacher** | **Strongly agree** | **Agree** | **Neutral** | **Disagree** | **Strongly disagree** |
| --- | --- | --- | --- | --- | --- | --- |
| 1 | has excellent clinical reasoning skills | 1 | 2 | 3 | 4 | 5 |
| 2 | conveys empathy for patients | 1 | 2 | 3 | 4 | 5 |
| 3 | communicates well with patients and relatives | 1 | 2 | 3 | 4 | 5 |
| 4 | understands learners’ needs and is committed to the growth of learners | 1 | 2 | 3 | 4 | 5 |
| 5 | establishes rapport with learners | 1 | 2 | 3 | 4 | 5 |
| 6 | has a positive attitude towards learners | 1 | 2 | 3 | 4 | 5 |
| 7 | demonstrates enthusiasm for his/her work | 1 | 2 | 3 | 4 | 5 |
| 8 | is patient | 1 | 2 | 3 | 4 | 5 |
| 9 | has a positive interaction with other health care workers | 1 | 2 | 3 | 4 | 5 |
| 10 | makes learning exciting and stimulating | 1 | 2 | 3 | 4 | 5 |
| 11 | has self-confidence | 1 | 2 | 3 | 4 | 5 |
| 12 | is available for learners | 1 | 2 | 3 | 4 | 5 |
| 13 | is honest and has integrity | 1 | 2 | 3 | 4 | 5 |
| 14 | has leadership qualities | 1 | 2 | 3 | 4 | 5 |
| 15 | is aware of his/her role model status | 1 | 2 | 3 | 4 | 5 |
| 16 | is nice and easy to work with | 1 | 2 | 3 | 4 | 5 |
| 17 | is professionally competent in difficult clinical situations and able to cope with adversity | 1 | 2 | 3 | 4 | 5 |
