# Supplementary material for: Role modelling in the training of hospital-based medical specialists: a validation study of the Role Model Apperception Tool (RoMAT)
Source: Perspect Med Educ. 2019 Jul 25;8(4):237–45. doi: 10.1007/s40037-019-00527-6 (PMC6684559; doi:10.1007/s40037-019-00527-6)
Supplement: Supplementary file 2 — Table 1: Characteristics of residents, clinical teachers and context [file 40037_2019_527_MOESM2_ESM.docx]

| **Table 1. Characteristics of residents, clinical teachers and context** | | | | |
| --- | --- | --- | --- | --- |
|  | | | | |
| ***Residents (N=187)*** | | | |  |
| Male/female * | |  | | 60/127 |
| Age (years) | |  | | 31.0 (28.0 – 33.0) |
| Specialty ^†^ | |  | |  |
| *Anesthesiology* | |  | 61 (32.6) |  |
| *Pediatrics* | |  | 26 (13.9) |  |
| *Internal medicine* | |  | 25 (13.4) |  |
| *Radiology and Nuclear medicine* | |  | 10 (5.3) |  |
| *Medical microbiology* | |  | 10 (5.3) |  |
| *Cardiology* | |  | 7 (3.7) |  |
| *Other ‡* | |  | 48 (25.8) |  |
| In training/not in training *^, §^ | |  | | 172/13 |
| Training year †^, §^ | |  | |  |
| *N/A* | |  | 13 (7.0) |  |
| *1* | |  | 36 (19.5) |  |
| *2* | |  | 39 (21.1) |  |
| *3* | |  | 30 (16.2) |  |
| *4* | |  | 40 (21.6) |  |
| *5* | |  | 22 (11.9) |  |
| *6* | |  | 5 (2.7) |  |
| Experience as a resident prior to current function (in months) | | | | 15.0 (6.0 – 26.5) |
|  | | | |  |
| ***Clinical teacher (N=35)*** | | | | |
| Male/female * | | | | 22/13 |
| Age in year groups † | | | |  |
| *<45* | | | 5 (14.3) |  |
| *45-49* | | | 8 (22.9) |  |
| *50-54* | | | 11 (31.4) |  |
| *55-59* | | | 5 (14.3) |  |
| *60-64* | | | 4 (11.4) |  |
| *≥65* | | | 2 (5.7) |  |
| Number of residents under guidance in groups † | | | |  |
| *<5* | | | 9 (25.7) |  |
| *5-10* | | | 10 (28.6) |  |
| *≥10* | | | 16 (45.7) |  |
|  | | |  |  |
| ***Context (N=187)*** | | | | |
| City † | | | |  |
| *Amsterdam* | | | 118 (63.1) |  |
| *Ede* | | | 13 (7.0) |  |
| *Utrecht* | | | 35 (18.7) |  |
| *Zwolle* | | | 21 (11.2) |  |
| Academic/General teaching | | | | 151/36 |
| Duration of resident-clinical teacher relationship (months) | | | | 18.0 (7.0 – 36.0) |
|  | | | |  |
| All values are expressed as median (IQR) unless specified otherwise  ^*^ Expressed as number of participants  ^†^ Expressed as number (proportion)  ^‡^ Includes multiple specialties (Clinical genetics, Dermatology, Gynecology, Hospital Pharmacy, Neurosurgery, Ophthalmology, Plastic surgery, Psychiatry, Radiotherapy, Rehabilitation Medicine, Rheumatology, Sports Medicine, Surgery, Urology)  ^§^ Two residents had a missing value for this question | | | | |
|  | | | | |

Desired position in text: in result section after subheading response.
